# Cognitive disengagement syndrome across attention deficit/hyperactivity disorder, autism spectrum disorder, and internalizing disorders: towards a transdiagnostic construct

**DOI:** 10.1007/s00787-026-03058-8

**Published:** 2026-05-22

**Authors:** Francesco Maria Boccaccio, Helen Klip, Madelon A. Vollebregt, Sara Pieters, Renata Rizzo, Concetta Pirrone, Jan K. Buitelaar

**Affiliations:** 1 Department of Educational Sciences, University of Catania, Catania, Italy; 2 Karakter Child and Adolescent Psychiatry, Nijmegen, The Netherlands; 3 Radboud University, Behavioural Science Institute, Nijmegen, the Netherlands; 4 Child and Adolescent Neurology and Psychiatric Section, Department of Clinical and Experimental Medicine, University of Catania, Catania, Italy; 5 Department of Medical Neuroscience, Donders Institute for Brain, Cognition and Behavior, Radboud University Medical Centre, Nijmegen, The Netherlands

**Keywords:** Cognitive disengagement syndrome, Attention deficit/hyperactivity disorder, Autism spectrum disorder, Internalizing disorders, Transdiagnostic construct, Sluggish cognitive tempo

## Abstract

Cognitive Disengagement Syndrome (CDS) refers to symptoms of fogginess, daydreaming, blank staring, and slowed behaviour. Growing evidence suggests that CDS reflects a transdiagnostic construct. This study aimed to examine (1) associations between age, sex, and CDS severity; (2) differences in CDS across Attention-Deficit/Hyperactivity Disorder (ADHD), Autism Spectrum Disorder (ASD), and internalizing disorders; and (3) associations between CDS and quality of life, as assessed by the KIDSCREEN-27. CDS was assessed in 958 clinically referred children and adolescents (mean age = 10.7 ± 2.8 years; 36.3% female) with a primary diagnosis of ADHD (*n* = 353), ASD (*n* = 494), or internalizing disorders (*n* = 111). Four Child Behaviour Checklist (CBCL) items (13, 17, 80, 102) were used to operationalize CDS. Analyses included ANOVAs, MANOVAs, and multiple regression analyses in the full sample and a subsample without comorbidities. CDS levels didn’t differ by sex, whereas adolescents (12—17 years) showed higher CDS levels than children (6—11 years). Across diagnostic groups, ASD (*M* = 2.6, SD = 1.8) and internalizing disorders (*M* = 2.6, SD = 1.9) displayed higher CDS scores than ADHD (*M* = 1.8, SD = 1.6; *p* < .001). These findings remained significant in the subsamples without comorbidities (*p* < .001). Multiple regression analyses indicated that higher CDS scores were significantly associated with lower quality of life (*B* = -2.20, *p* < .001), independent of diagnostic group, sex, and age, without significant interaction between CDS and diagnosis. Correlational analyses indicated stronger associations between CDS with autistic (*r* = .25-.36; *p* < .05) and affective CBCL symptom dimensions (*r* = .19-.36; *p* < .05) dimensions than with ADHD symptoms. CDS appears to represent a clinically relevant transdiagnostic construct. Its strong expression in ASD suggests that it captures disengagement mechanisms beyond current nosological frameworks.

**Supplementary Information:**

The online version contains supplementary material available at https://doi.org/10.1007/s00787-026-03058-8.

## Introduction

Cognitive Disengagement Syndrome (CDS), previously known as Sluggish Cognitive Tempo (SCT), refers to a cluster of persistent and developmentally inappropriate behaviours, characterized by both cognitive and motor features. The cognitive features encompass a reduced connection between attention, conscious effort, and the external environment—often observed as excessive staring, frequent daydreaming, mental confusion or fogginess, withdrawal, and a sleepy appearance. Motor features, on the other hand, are typically marked by low activity levels, long periods of passivity or sedentary behaviour, and movements that are slow, limited, or delayed [1]. More specifically, research has identified fifteen behaviours associated with CDS, which can be grouped into three main categories: daydreaming, mental confusion, and hypoactivity [2].

Initially, most research focused primarily on CDS within the context of Attention Deficit/Hyperactivity Disorder (ADHD), particularly the inattentive presentation, and highlighted a strong association with inattentive symptoms [3]. Later studies have demonstrated that CDS symptoms are empirically distinct from those of ADHD inattention [4–15]. Moreover, evidence further suggests that CDS is related to internalizing dimensions, especially with depression and anxiety [1, 16–19], but also to autism [20–23]. Regarding the latter, the findings suggest that CDS traits may directly impact functional outcomes in autistic children [24], and may also constitute a childhood risk factor for autism in adolescence [25].

Although CDS has been most consistently linked to internalizing symptoms, emerging evidence suggests that its associations may extend beyond internalizing dimensions. Although CDS has been most consistently linked to internalizing symptoms, emerging evidence suggests that its clinical correlates may extend beyond internalizing dimensions. Some studies have reported relationships between CDS and externalizing symptoms, including behavioural dysregulation and oppositional features, albeit generally weaker and less consistent than those observed for anxiety and depression [26]. Additionally, these latter associations typically remain significant when controlling for ADHD symptoms [27]. Moreover, there is evidence that CDS is also further linked to broader functional impairments [4, 23, 28, 29]. In line with this, higher CDS scores are also associated with lower psychological well-being and reduced quality of life [12, 30], although research examining the relationship between CDS and quality of life remains limited.

Together, these findings highlight the clinical relevance of CDS across diagnostic contexts. This broad pattern of associations raises an important question about how CDS should be conceptualized. At the same time, several conceptual questions about CDS remain unresolved. Specifically, it is still debated whether CDS should be regarded as a *diagnostic specifier*, a *transdiagnostic construct*, or an *independent psychopathological entity* [31]. Clarifying these alternatives is crucial, as each conceptualization carries distinct implications for diagnosis, research, and treatment. Within current diagnostic systems, for example, *specifiers* are used as extensions of a diagnosis that add information on the course, severity, or distinctive clinical features of a disorder, thereby allowing for more precise case formulation and tailored interventions [32]. These differ from *subtypes*, which instead define mutually exclusive and jointly exhaustive subgroups within a diagnosis; unlike subtypes, specifiers are not mutually exclusive, and more than one can apply simultaneously [32]. If CDS is conceptualized as a *transdiagnostic construct*, this would imply that it represents a process or trait occurring across different mental disorders, functioning either as a vulnerability factor or as a maintaining mechanism [33]. Such transdiagnostic processes are commonly found in cognitive, emotional, and behavioural domains, such as attention, memory, reasoning, and regulation, and may contribute to explaining high rates of comorbidity as well as guiding the development of interventions that target shared mechanisms. This dimensional and process-oriented perspective aligns closely with the Research Domain Criteria (RDoC) framework proposed by the National Institute of Mental Health, which emphasizes the study of mental functions across multiple levels of analysis, from genes to behaviour, rather than within traditional categorical boundaries [34].

In parallel, hierarchical models of psychopathology provide an additional framework for situating CDS within the broader structure of mental disorders. A growing body of evidence suggests that psychopathology is organized hierarchically, with a general factor (p factor) accounting for shared variance across disorders, alongside higher-order internalizing and externalizing dimensions. Within this framework, emerging findings indicate that, although CDS is strongly correlated with ADHD inattention, it shows unique associations with internalizing symptoms when controlling for ADHD-IN, whereas ADHD-IN is more strongly linked to externalizing symptoms [3]. This pattern has led to the hypothesis that different attentional dimensions may occupy distinct positions within the hierarchical structure of psychopathology, with CDS potentially aligning more closely with internalizing liability. Such a perspective may help clarify both the overlap and differentiation between CDS and ADHD-related constructs, as well as their role in the co-occurrence of internalizing and externalizing difficulties [35]. Framing CDS within an RDoC perspective could therefore facilitate a more nuanced understanding of its underlying neurocognitive mechanisms and its transdiagnostic role across psychopathologies. Finally, considering CDS as an *independent psychopathological entity* would imply that it holds sufficient distinctiveness and internal coherence to justify its recognition as a separate diagnostic category, a step that carries important implications both for nosology and for clinical practice. However, further research is needed to determine whether and how CDS symptomatology is present across psychopathologies in order to advance the conceptualisation of CDS.

Although CDS has been observed across a range of disorders, direct comparisons between clinical groups remain limited. Such comparisons are necessary to determine whether CDS reflects disorder-specific symptomatology or a broader transdiagnostic dimension. In light of this evidence, determining whether CDS is specific to particular diagnostic categories or represents a transdiagnostic dimension is essential for clarifying its conceptual status and informing clinical assessment. Moreover, while CDS has been linked to functional impairments and internalizing symptoms, evidence regarding its impact on broader indicators of well-being, including quality of life, is limited. Investigating these associations is critical to understanding the practical significance of CDS in daily functioning and psychological health. Finally, as most prior studies have focused on community samples or ADHD-referred samples, the present study extends this work to a large clinical sample referred to an expert centre for child and adolescent psychiatry, a sample unselected for CDS and thus providing a unique opportunity to examine CDS in a broader clinical context. The choice to select ADHD, ASD, and internalizing disorders in the present study is theoretically and empirically grounded. ADHD provides an essential reference point, as CDS has historically been studied most extensively in relation to ADHD. Autism, in contrast, is characterized by atypical patterns of attentional engagement, social withdrawal, and reduced responsiveness to environmental stimuli, which conceptually overlap with core features of CDS such as disengagement and mental fogginess. Internalizing disorders, including anxiety and depression, have consistently shown robust associations with CDS symptoms, particularly in relation to low energy, and cognitive slowing.

Taken together, these three diagnostic domains capture distinct yet complementary manifestations of attentional and cognitive disengagement—spanning neurodevelopmental, socio-cognitive, and affective dimensions. As such, provide a theoretically meaningful framework to examine CDS as a potential transdiagnostic construct rather than a disorder-specific phenomenon.

In addition, investigating demographic influences such as sex and age is essential, as existing studies have produced inconsistent results: some report higher CDS levels in males [36], others in females [37], while several find no significant differences [38, 39]. Understanding whether these differences reflect true sex-related patterns or sample-specific variations can clarify the generalizability of CDS findings. Moreover, examining age-related changes provides insight into its developmental trajectory, since prior research suggests CDS symptoms may become more pronounced in adolescence [3].

The present study had three primary aims: (1) to examine the role of demographic factors (sex and age) on CDS severity within and across diagnostic groups; (2) to compare CDS symptom expression across children and adolescents with clinical consensus diagnoses of ADHD, ASD, and internalizing disorders in order to evaluate its potential transdiagnostic nature and to account for the potential influence of comorbidities, which were subsequently addressed through sensitivity analyses; and (3) to investigate the functional relevance of CDS by testing its association with quality of life (KIDSCREEN-27) and its associations with DSM-oriented, data-driven CBCL scales, both in the full samples and in subsamples without comorbidities.

## Methods

### Procedures

In this study, data were derived from a clinical database of referrals to Karakter Child and Adolescent Psychiatry. Karakter is an academic expert centre for child and adolescent psychiatry in the Netherlands, providing specialised outpatient and inpatient care for children and adolescents with various mental health conditions. As part of usual clinical routine, prior to their first appointment, parents were asked to complete a digital intake questionnaire, the Child Behaviour Checklist (CBCL/6–18) [40], and the KIDSCREEN-27 questionnaire [41].

All data used in this study were obtained prospectively. Cases were pseudonymised (e.g., personal identifiers such as names and addresses were removed), ensuring that no individual health records were directly assessed and that privacy was maintained. This study was not subject to the Dutch Medical Research Involving Human Subjects Act (WMO). This was confirmed by the Medical Ethics Review Committee of Radboud University Medical Center (RUMC) [42]. Patients and their legal guardians were informed that data collected during routine care could be used for scientific research. They could opt out of having their data used for research purposes at any time, with the assurance that this decision would not affect their treatment.

### Participants

Data were extracted between July 2018 and July 2025 by an authorised data manager. Only cases with the following data available were included: (1) age (≥ 6 years), (2) sex, (3) clinical DSM-5 diagnoses of Attention Deficit/Hyperactivity Disorder (ADHD), Autism Spectrum Disorder (ASD), and internalizing disorders (combination of anxiety [*n* = 25], depression [*n* = 13], and post-traumatic stress disorder [PTSD, *n* = 73]) as the primary diagnosis, (4) other axis-I conditions as comorbidities, (5) CBCL/6–18 items, and (6) KIDSCREEN-27 subscale scores.

Anxiety, depression and PTSD were grouped together in internalizing disorders in line with established dimensional models of psychopathology, in which these conditions load on a higher-order internalizing factor characterized by shared affective and emotional processes [35, 40]. Moreover, in our sample, the number of participants within each of these categories was relatively small compared to the ADHD and ASD groups. Further subdivision would therefore have resulted in very small subgroups, substantially reducing statistical power and limiting the reliability and interpretability of the findings.

Clinical DSM-5 diagnoses were established by a multidisciplinary team consisting of a mental health specialist (child and adolescent psychiatrist or clinical psychologist/clinical neuropsychologist) and a mental health generalist (nurse practitioner or psychologist). Diagnoses were based on comprehensive clinical evaluations, including developmental history, clinical interviews with caregivers and the child, behavioural observations, and information collected during routine assessment. Although no standardized structured diagnostic interview was systematically administered across all cases, the diagnostic process followed standard clinical practice within a specialized tertiary care setting.

An a priori power analysis for a one-way ANOVA (fixed effects, omnibus; effect size *f* = 0.25; *α* = 0.05; 1 − β = 0.95; 3 groups) indicated a required total sample of *N* = 252 (critical *F* = 3.03; *λ* = 15.75; numerator df = 2; denominator df = 249). Our final sample (*N* = 958) and the no-comorbidity sample (*N* = 296) therefore exceeded this requirement.

The final sample consisted of 958 children and adolescents (36.4% female; mean age = 10.7 ± 2.8 years) (see Table 1). Given the high rates of comorbidity in clinical populations, many participants also met criteria for additional disorders. To account for this, a subsample without comorbid diagnoses was created (*n* = 296; 37.5% female; mean age = 10.9 ± 2.8 years), including only individuals who did not receive any additional diagnoses across repeated clinical assessments.

**Table 1:** Demographic and clinical features of the full and no-comorbidity samples

	*Female*			*Male*			*Total*		
	N	%	Mean age (SD)	N	%	Mean age (SD)	N	%	Mean age (SD)
Full sample									
ADHD	108	11.2%	10.7 (2.9)	245	25.5%	10.2 (2.8)	353	36.7%	10.3 (2.8)
ASD	177	18.4%	11.4 (2.6)	317	33.0%	10.4 (2.8)	494	51.5%	10.7 (2.8)
Internalizing disorders	63	6.6%	12.4 (2.7)	48	5.0%	10.0 (2.7)	111	11.5%	11.4 (2.9)
Total	348	36.3%	11.3 (2.8)	610	63.7%	10.3 (2.8)	958	100%	10.7 (2.8)
No-comorbidity sample									
ADHD	41	18.8%	11.4 (2.7)	87	29.4%	10.3 (2.7)	128	43.2%	10.7 (2.7)
ASD	54	18.2%	11.1 (2.8)	84	28.4%	10.3 (2.8)	138	46.6%	10.7 (2.8)
Internalizing disorders	16	5.4%	12.8 (2.9)	14	4.7%	10.2 (2.7)	30	10.1%	11.6 (2.9)
Total	111	37.5%	11.6 (2.8)	185	62.5%	10.3 (2.7)	296	100.0%	10.9 (2.8)

### Measurements

#### Child Behavior Checklist (CBCL/6—18)

The Child Behavior Checklist (CBCL/6–18) is a widely used caregiver-report instrument with 113 items designed to assess emotional and behavioural problems in children and adolescents aged 6 to 18 years. Each item is rated on a 3-point scale (0 = not true, 1 = somewhat or sometimes true, 2 = very true or often true), reflecting how accurately each statement describes the child’s behaviour. The scores include eight problem scales: withdrawn (1); somatic (2); anxious (3); social (4); thought (5); attention (6); rule-breaking (7); aggressive (8); and other problems. The sum of the problem scale 1,2 and 3 form the scale ‘internalizing behaviour’; 7 and 8 form ‘externalizing behaviour’. All subscales together count for the total problem scale. Some items contribute to more than one problem scale. T scores are computed from raw scores; higher scores on the syndrome scales indicate greater severity of problems. A T score of 63 (90th percentile) demarcates the clinical range, indicating that a child may require professional help [40]. The CBCL/6–18 has well-established psychometric properties in clinical, non-clinical, and cross-cultural populations [43–45].

To describe the diagnostic groups in a dimensional way, items belonging to the following DSM-oriented, data-driven scales were included: Affective problems (items 5, 14, 18, 24, 35, 52, 54, 76, 77, 91, 100, 102, 103; Cronbach’s alpha 0.78), Anxiety problems (items 11, 29, 30, 45, 50, 112; Cronbach’s alpha 0.76), ADHD (items 4, 8, 10, 41, 78, 93, 104; Cronbach’s alpha 0.81), and ASD traits (items 1, 25, 29, 42, 46, 66, 79, 84, 111; Cronbach’s alpha 0.66) [46]. We further subdivided the ADHD scale into ADHD Inattention (ADHD_I) (items 4, 8, 78; Cronbach’s alpha 0.74) and ADHD hyperactivity/impulsivity (ADHD_H) (items 10, 41, 93, 104; Cronbach’s alpha 0.77).

#### Cognitive Disengagement Syndrome (CDS)

Several instruments have been developed to measure CDS [2], but they are still rarely used in large clinical samples, limiting systematic comparisons across disorders and populations. Here, we adopt the operationalization of CDS using four items from the CBCL/6—18 as applied by previous studies [16, 47, 48]. This approach provides a standardized and replicable way to capture core CDS behaviours across diverse clinical groups. CDS was operationalized using four items of CBCL/6—18: *item 13—confused or seems to be in a fog*, *item 17—daydreams or gets lost in his/her thoughts*, *item 80—stares blankly*, and *item 102—underactive, slow moving, or lacks energy* (Cronbach’s alpha in this study was 0.57) [40].

In the present study, we used two different sets of CBCL-CDS items: the full set of four items (CBCL-CDS-4) and a reduced set of three items (CBCL-CDS-3), excluding item 102, as it overlaps with the affective problems DSM-oriented scale. Also in this case, Cronbach’s alpha was 0.57. Given the strong association between CDS and internalizing disorders, we repeated all analyses with both items sets to ensure that our findings reflect CDS specifically and are not confounded by overlapping behaviours.

#### KIDSCREEN-27 questionnaire

The KIDSCREEN-27 questionnaire was used to assess the quality of life in children and adolescents, based on parent reports [49]. This instrument evaluates five domains: Physical Well-being (ICC 0.65), Psychological Well-being (ICC 0.64), Autonomy and Parent Relations (ICC 0.66), Peers and Social Support (ICC 0.61), and School Environment (ICC 0.74) [41]. All items were rated on a 5-point Likert scale, ranging from 1 (not at all/never) to 5 (totally true/always). Lower scores on individual subscales or the overall sum score indicate lower subjective health and well-being.

### Statistical analysis

All statistical analyses were conducted using R Studio (Version 4.2; [50]) and IBM SPSS Statistics (Version 30; [51]). Within R, the following packages were used: ggplot2 [52], dplyr [53], rstatix [54], patchwork [55], corrplot [56], and cocor [57].

Independent-samples t-tests were conducted to examine differences in CBCL-CDS-4 and CBCL-CDS-3 scores by sex (male vs. female) and by age group (children aged 6–11 years vs. adolescents aged 12–18 years). Group differences on CBCL-CDS-4 and CBCL-CDS-3 scores across diagnostic categories (ADHD, ASD, internalizing disorders) were assessed using Welch’s one-way ANOVA followed by Games–Howell post hoc tests, chosen to accommodate heterogeneity of variances and unequal sample sizes. To account for demographic effects, MANCOVAs on the same outcomes were performed with sex and age entered as covariates, and Bonferroni-adjusted post hoc tests were applied when multivariate effects were significant. All analyses were first conducted on the full clinical sample and subsequently repeated in a subsample without comorbidities to perform sensitivity analyses and control for diagnostic confounding.

A multiple linear regression analysis was conducted to examine whether CBCL-CDS-4 scores were associated with children’s quality of life (KIDSCREEN). In the model, we included diagnostic group (ADHD, ASD, internalizing), sex, and age as covariates, in order to control for their potential effects on the outcome variable. Models were repeated using CBCL-CDS-3 scores to assess the impact of excluding the overlapping item.

Correlations between individual CBCL-CDS items, CBCL-CDS-3 and DSM-oriented CBCL scale scores (ADHD, ADHD_I, ADHD_H, Affective, Anxiety, and ASD Problems) were computed and visualized. Steiger’s tests for dependent correlations were applied to formally compare coefficients, providing item-level evidence on the convergent and discriminant validity of CDS within the CBCL framework.

Finally, associations between cognitive disengagement syndrome (CDS) symptoms and the number of comorbidities were examined using Poisson regression models, given the count nature of the outcome variable. CDS was first entered as a continuous variable to test for a linear association with comorbidity burden. To explore potential non-linear or threshold effects, CDS was also analysed as a categorical variable by grouping scores into four levels (0, 1–2, 3–4, 5–6), with the CDS = 0 group as the reference category. Results are reported as regression coefficients (β), standard errors (SE), incidence rate ratios (IRR), and corresponding 95% confidence intervals (CI). For all analyses, statistical significance was set at *p* < 0.05.

## Results

Independent samples t-tests for each diagnostic group showed no significant sex differences in CBCL-CDS-4 or CBCL-CDS-3 in the full sample, except in the internalizing no-comorbidity group, where females scored higher on CBCL-CDS-4 than males (Table S1). Adolescents scored higher than children on CBCL-CDS-4 across all diagnostic groups in both the full sample and the no-comorbidity subsample, with the exception of the internalizing disorders group in the latter (Table S1).

**Fig. 1:**
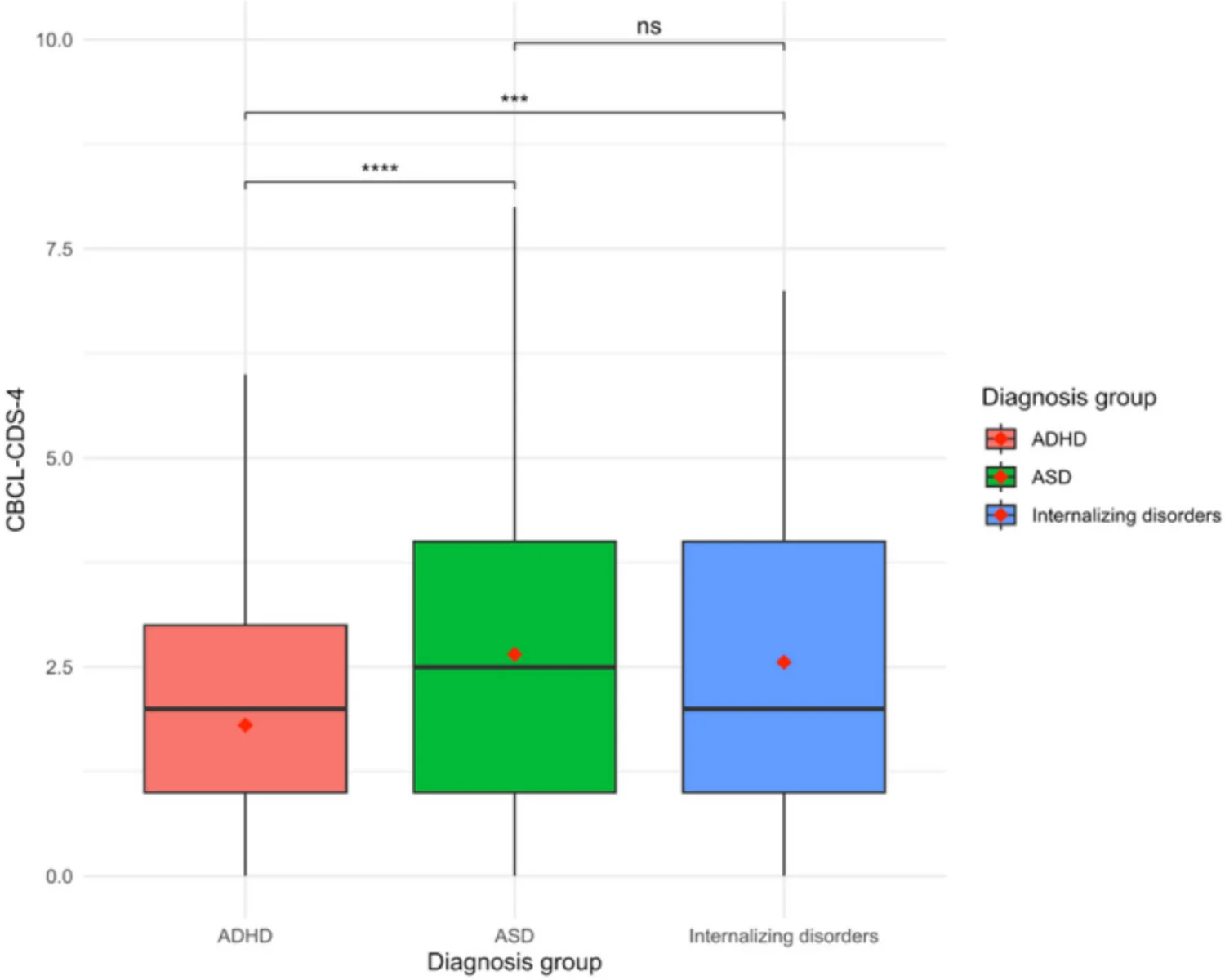
Full sample Welch’s ANOVA. *Note.* Boxplots of CBCL-CDS-4scores across the three diagnostic groups (ADHD, ASD, and internalizing disorders). Red diamonds represent group means, while thick black lines within boxes denote medians. Boxes illustrate interquartile ranges (IQR), and whiskers indicate data ranges excluding outliers. Group comparisons were conducted using Welch’s ANOVA (*p* < 0.001 for both panels), followed by Games-Howell post hoc tests. **** *p* < 0.0001; *** *p* < 0.001; ** *p* < 0.01; * *p* < 0.05; ns = not significant

Welch’s ANOVAs in the full sample revealed significant diagnostic group differences for CBCL-CDS-4 (Table S2), with ASD (*M* = 2.7, SD = 1.8) and internalizing disorders (*M* = 2.6, SD = 1.9) scoring higher than ADHD (*M* = 1.8, SD = 1.6), while ASD and internalizing disorders did not differ significantly from each other (Fig. 1; Table S3). The results remained consistent for CBCL-CDS-3 and remained significant when sex and age were included as covariates.

The same results pattern was replicated in the no-comorbidity subsample (Figure S1, Table S2 and S3), with the exception of the internalizing disorders group without comorbidity, which showed no significant differences. The same pattern of statistical significance was observed for CBCL-CDS-3 and when sex and age were included as covariates.

**Fig. 2:**
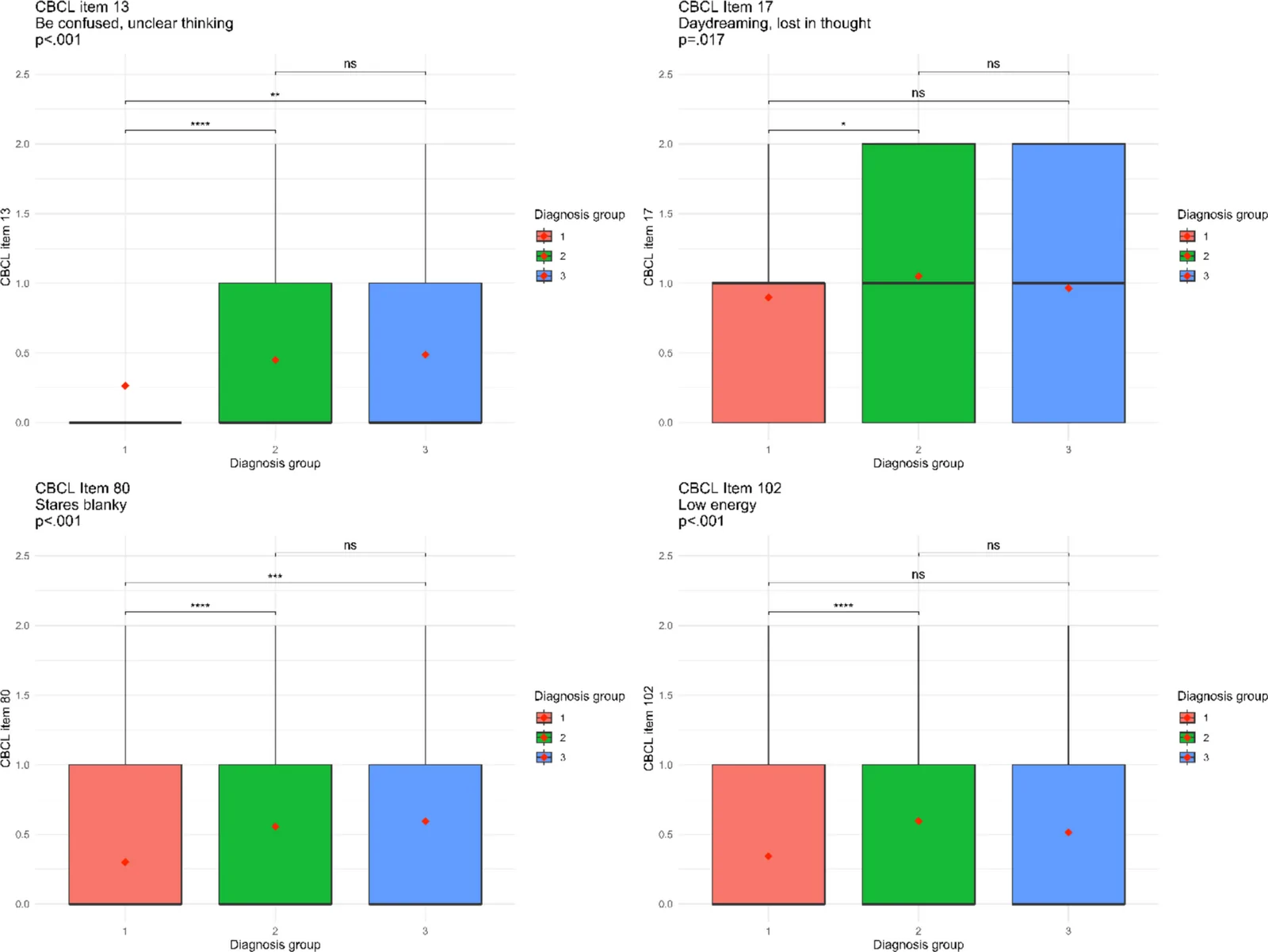
Full sample Welch’s ANOVA single CBCL-CDS items. *Note:* Boxplots of CBCL-CDS items scores across the three diagnostic groups (ADHD, ASD, and Internalizing disorders). Red diamonds represent group means, while thick black lines within boxes denote medians. Boxes illustrate interquartile ranges (IQR), and whiskers indicate data ranges excluding outliers. Group comparisons were conducted using Welch’s ANOVA, followed by Games-Howell post hoc tests. **** *p* < 0.0001; *** *p* < 0.001; ** *p* < 0.01; * *p* < 0.05; ns = not significant

Next, we explored whether the group differences observed for the CBCL-CDS-4 constructs were consistent at the level of individual CDS items. Item-level Welch’s ANOVAs showed significant group effects for item *13 – confused/unclear thinking*, *item 80 – stares blankly*, *item 17 – daydreaming/lost in thought*, and *item 102 – not very active/low energy* (Table S2). Post hoc Games–Howell tests indicated that the ASD group scored higher than the ADHD group on all four items, while the internalizing group also scored higher than ADHD on items 13 and 80 (Fig. 2; Table S3). The same pattern of statistical significance was observed for CBCL-CDS-3 and when sex and age were included as covariates.

All item-level results were replicated in the no-comorbidity subsample (Tables S2 and S3). Specifically, Welch’s ANOVAs showed significant group effects for *item 13—confused/unclear thinking*, *item 80—stares blankly*, and *item 102—not very active/low energy*, while no effect emerged for *item 17—daydreaming/lost in thought* (Table S2). Post hoc Games–Howell tests indicated that the ASD group scored higher than the ADHD group on items 13, 80, and 102, and that the internalizing group also scored higher than ADHD on item 80 (Figure 2S, Table S3). The same pattern of statistical significance was observed when sex and age were included as covariates.

Next, we explored the association between CDS and quality of life. The regression showed that higher CDS scores were significantly associated with lower KIDSCREEN-27 scores (see Table 2). Compared with the ADHD group, children with ASD and those with internalizing disorders reported lower levels of quality of life. Sex and age were also significant covariates: girls had slightly higher KIDSCREEN-27 scores, while older age was associated with lower scores. The interaction between CDS and diagnostic group was not significant, indicating that the association between CDS and quality of life did not differ across diagnostic groups. The same analysis was repeated with CBCL-CDS-3, yielding the same results.

**Table 2:** Multiple linear regression predicting kidscreen-27 total score from cbcl-cds-4, diagnosis group, sex, and age (full sample and no-comorbidity sample)

	Full sample				No-comorbidity sample			
Predictor	B	SE	t	*p*	B	SE	t	*p*
Intercept	112.04	2.15	52.15	** < 0.001**	113.85	3.83	29.76	** < 0.001**
CBCL-CDS-4	−2.20	0.37	−5.93	** < 0.001**	−2.32	0.60	−3.87	** < 0.001**
ASD	−2.59	1.25	−2.08	**0.038**	−6.93	2.20	−3.15	**0.002**
Internalizing	−4.65	1.99	−2.34	**0.020**	−7.94	3.47	−2.29	**0.023**
Sex	1.53	0.76	2.01	**0.045**	1.88	1.34	1.40	**0.162**
Age	−1.10	0.13	−8.43	** < 0.001**	−1.02	0.23	−4.42	** < 0.001**
CBCL-CDS-4 × ASD	−0.16	0.46	−0.35	0.724	0.30	0.78	0.38	0.705
CBCL-CDS-4 × Internalizing	0.15	0.67	0.22	0.828	0.18	1.06	0.17	0.863

**Fig. 3:**
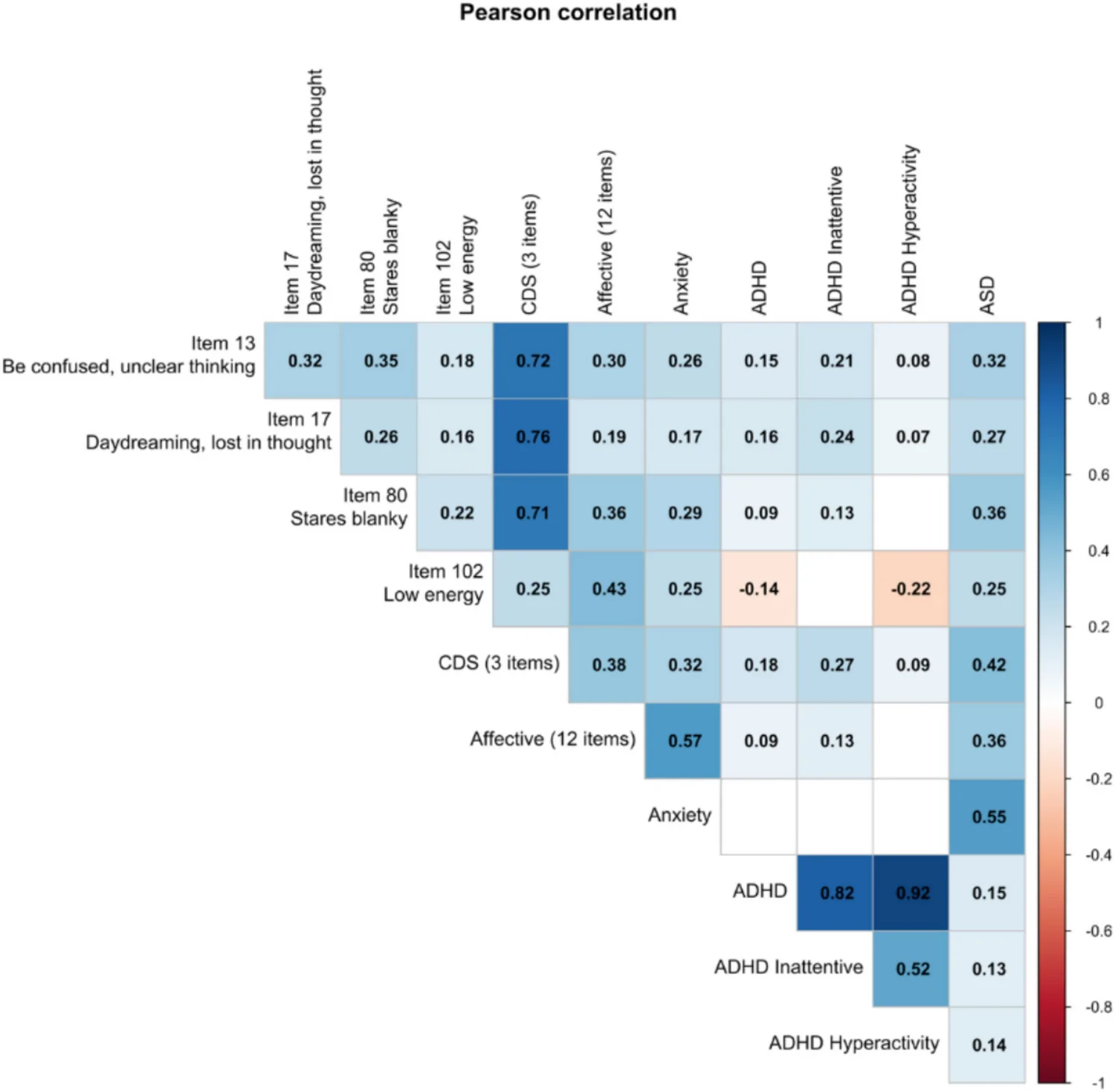
Pearson’s correlation matrix among CBCL-CDS items (13, 17, 80, 102), CBCL-CDS-3 and CBCL DSM-oriented scales. *Note.* Blue indicates positive and red negative correlations; colour intensity reflects magnitude. Coloured cells indicate a significant correlation (*p* < 0.05)

Moreover, we explored the associations of the CDS construct with other relevant CBCL scales, moving beyond categorical diagnoses. Correlation analyses indicated that all four CBCL-CDS items, as well as the CBCL-CDS-3 subscales were significantly more strongly associated with ASD and with Affective Problems (12-item version) than with ADHD Inattention (ADHD_I) and ADHD hyperactivity/impulsivity (ADHD_H), with Steiger’s tests confirming these differences (Fig. 3; Table S4).

Finally, associations between CDS symptoms and the number of comorbidities were examined using Poisson regression models (Table 3). CDS was first analysed as a continuous variable. To explore potential non-linear or threshold effects, CDS was also categorized into four groups (0, 1–2, 3–4, 5–6). CDS scores were not significantly associated with the number of comorbidities (*β* = −0.005, SE = 0.015, *p* = 0.74). Consistent results were observed when CDS was analysed as a categorical variable, with no significant differences in the number of comorbidities across CDS groups (all *p* > 0.48).

**Table 3:** Association between CDS and number of comorbidities (Poisson regression)

Predictor	β (SE)	IRR (95% CI)	*p*-value
CDS (continuous)	−0.005 (0.015)	0.995 (0.966–1.025)	0.74
CDS group			
1–2 vs 0	−0.040 (0.058)	0.961 (0.858–1.076)	0.49
3–4 vs 0	−0.038 (0.066)	0.963 (0.846–1.096)	0.57
5–6 vs 0	−0.044 (0.107)	0.957 (0.775–1.181)	0.68

## Discussion

This study investigated the presence and profile of CDS symptoms across different clinical populations, namely ADHD, ASD, and internalizing disorders. The first aim was to examine the role of demographic factors, such as sex and age, in CDS severity within and across diagnostic groups. The absence of sex effects across most groups, except for the internalizing group without comorbidities, suggests that sex exerts only a marginal or context-specific influence. This finding aligns with previous literature, where evidence on sex differences in CDS remains inconsistent: some studies reported higher CDS levels in males [36], others in females [37, 58, 59], while several found no significant differences at all [38, 39, 60, 61]. In contrast, our finding of an age-related increase in CDS symptoms, particularly within ADHD and ASD, suggests that adolescence may represent a developmental period in which mental slowness, daydreaming, and confused thinking become more prominent. This result is consistent with prior evidence. For instance, Jiang et al. (2024), in a large sample of 72,106 Chinese students, found stronger associations of cognitive symptom nodes in adolescents compared to children [48]. Other studies similarly reported that CDS symptoms increase with age [3, 11]. This age-related increase may reflect developmental changes in executive and attentional control systems, which undergo significant restructuring during adolescence. As cognitive demands and environmental expectations intensify, difficulties in sustaining attention, processing speed, and cognitive flexibility may become more apparent [62]. Moreover, increased self-awareness and metacognitive capacity during adolescence may enhance the subjective perception of mental slowness and disengagement, contributing to higher reported CDS symptoms [63].

The second aim was to compare CDS symptom expression across children and adolescents with ADHD, ASD, and internalizing disorders, to evaluate its potential transdiagnostic nature. Group comparisons, consistent across both the CBCL-CDS-4 and the reduced CBCL-CDS-3, and in both the full and no-comorbidity samples, indicated that children with ASD or internalizing disorders exhibited higher CDS levels than those with ADHD. This pattern was further supported by correlation analyses using CBCL DSM-oriented groups, where all CDS items showed stronger associations with ASD and internalizing symptoms than with ADHD. This finding is in line with studies highlighting the unique association of CDS with internalizing disorders, especially depression, where higher CDS scores were associated with higher depressive symptoms controlling for ADHD [5–9, 14, 19, 36, 64–70]. Similarly, other evidence suggests that individuals with ASD display elevated CDS symptoms independently of ADHD [22, 23]. At the same time, prior research has emphasized that CDS is not reducible to internalizing disorders [16, 17, 37] nor to autism [20].

Our item-level analyses further clarified these associations: the ASD group scored higher than ADHD on all four CDS items, except for *item 17—daydreams or gets lost in his/her thoughts* in the no-comorbidity subsample. This pattern suggests that “*confused thinking*”, “*stares blankly*”, and “*not very active/low energy*” may be more typical of ASD than ADHD. Correlation analyses supported this interpretation, showing that CDS was more strongly associated with autistic traits than with ADHD (both ADHD_I and ADHD_H). Rather than confirming models that position ADHD as the central framework for inattention and sluggishness, these findings point to CDS as a distinct construct characterized by cognitive disengagement, mental fog, and rumination, closely tied to autistic features. This distinction is particularly relevant given the frequent comorbidity between ASD and ADHD, often attributed to shared inattentive symptoms that may, in fact, reflect qualitatively different mechanisms. Previous research proposed that the overlap between ASD and ADHD traits is primarily explained by shared attention-related difficulties, including inattention and deficits in attentional switching [71]. Yet emerging evidence suggests that such attentional problems differ across disorders: while in ADHD they are largely driven by inhibition deficits and distractibility, in ASD they may reflect a tendency to hyperfocus, or problems in social attention or sensory processing peculiarities that interfere with responding to complex cues [72]. From this perspective, inattentive symptoms in ASD may not truly overlap with ADHD-related inattention, but instead align more closely with the phenomenology of CDS, where disengagement from the social context is central and manifests in greater social withdrawal and difficulties initiating or joining activities [64, 73, 74]. This conceptual alignment reinforces the need to reconsider how inattentive symptoms are defined and interpreted across ASD and ADHD.

The third aim was to assess the functional relevance of CDS by examining its association with quality of life (KIDSCREEN-27) and with DSM-oriented, data-driven CBCL scales, both in the full sample and in subsamples without comorbidities. Regression analyses indicated that CBCL-CDS-4 scores were negatively associated with quality of life across all diagnostic groups, with no significant interaction between CDS scores and diagnostic group. These findings are consistent with prior studies showing that higher CDS scores were associated with lower psychological well-being, reduced autonomy and parent–child relations, and diminished peer and social support [9, 12, 75]. These findings add important information to the scientific literature, as research on the impact of CDS on broader indicators of well-being, including quality of life, is still limited. Importantly, the results highlight the clinical relevance of considering CDS in diagnostic and functional evaluations, as it improves the prediction of well-being beyond traditional categorical diagnoses.

Interestingly, despite previous evidence [7], in this study CDS scores were not significantly associated with the number of comorbidities, whether analysed continuously or categorically. This indicates that CDS is not merely a marker of general clinical severity or total comorbidity load, but rather a distinct dimension of cognitive disengagement and mental slowness that can manifest across diagnostic groups. Clinically, this highlights the importance of assessing CDS even in youth with relatively few comorbid conditions.

### CDS conceptual framework and further directions

A central question is whether CDS should be conceptualized as an independent construct, a transdiagnostic dimension, or a diagnostic specifier. Evaluating CDS as an independent entity would require population-based studies identifying individuals with CDS-only profiles and assessing whether they experience meaningful functional impairment. In this respect, the framework proposed by Robins and Guze (1970) offers a benchmark for evaluating whether CDS qualifies as an independent diagnostic entity [76].

A valid category should show a coherent clinical profile, clear boundaries from other disorders, familial aggregation, biological correlates, and longitudinal stability. Establishing CDS as independent would thus require epidemiological evidence, distinctiveness from existing DSM diagnoses, and predictive validity over time. If CDS-only profiles are rare or not associated with significant impairment, it may instead function as a transdiagnostic factor that amplifies or maintains difficulties across ADHD, ASD, or internalizing disorders. This is supported by evidence that CDS symptoms consistently relate to internalizing problems, social withdrawal, and autistic traits across diagnostic boundaries [1]. Our findings are consistent with this view: CDS levels were higher in ASD and internalizing groups than in ADHD, illustrating the cross-cutting expression of CDS. Future research should use longitudinal designs and advanced statistical models to clarify whether CDS predicts symptom escalation, functional decline, or comorbidity. Intervention studies could also explore whether targeting CDS-related processes improves outcomes across disorders.

Finally, considering CDS as a diagnostic specifier within ADHD, ASD, or mood/anxiety disorders may offer clear clinical utility by highlighting by individuals with additional “foggy” features and potentially distinct prognosis or treatment response. However robust evidence is required to justify CDS as a specifier, and prospective studies assessing CDS at baseline are required to evaluate long-term outcomes and treatment responses across subgroups.

### Limitations

Despite this study being conducted in a large representative clinical sample of children and adolescents with various mental health conditions, some limitations should be considered when interpreting these findings. First, diagnoses were based on multidisciplinary clinical consensus rather than standardized structured diagnostic interviews. Although this approach reflects routine clinical practice and enhances ecological validity, it may limit comparability with studies using fully standardized diagnostic procedures and may introduce variability in diagnostic classification. Second, the internalizing no-comorbidity group was relatively small, reducing statistical power and potentially limiting the detection of age or sex effects within this subgroup. Third, all measures of CDS were derived from the CBCL, which, although widely used and psychometrically sound, relies on parent report and may not fully capture the phenomenology of CDS as experienced by youth or observed in other contexts. Fourth, the cross-sectional design precludes conclusions about developmental trajectories or causal pathways linking CDS with internalizing symptoms and autistic traits. Finally, although comorbidity was statistically controlled, diagnostic classifications were based on consensus clinical assessment and may still reflect heterogeneity within groups.

## Conclusion

Although the present study cannot resolve broader nosological debates [31], its findings provide converging support that CDS represents a transdiagnostic dimension. Higher CDS scores in ASD and internalizing groups compared to ADHD, the persistence of these differences in no-comorbidity subsamples, and the consistent prediction of poorer quality of life across diagnoses suggest that CDS is a cross-cutting process rather than a feature restricted to a single disorder. These results do not preclude the possibility that CDS may also function as an independent construct or serve as a clinically useful specifier.

CDS extends beyond ADHD-related inattention and is strongly present in ASD, reflecting mechanisms such as mental fog, blank staring, and slowed behavior that are not captured by current diagnostic systems. It predicts lower quality of life and differentiates clinical groups beyond comorbidities, highlighting its clinical relevance. Recognizing CDS may improve understanding of overlaps among ADHD, ASD, and internalizing disorders and support mechanism-based interventions. Future longitudinal, multi-informant, neurocognitive, and neurobiological studies are needed to clarify its developmental trajectory, underlying mechanisms, and potential for targeted interventions. Systematic assessment of CDS could enhance early detection and personalized treatment, ultimately improving youth functioning and well-being.

## Supplementary Information

Below is the link to the electronic supplementary material.

Supplementary file1 (DOCX 223 KB)

## Data Availability

Data are available upon request from Dr. Helen Klip (h.klip@karakter.com).
