# Supplementary material for: Cognitive disengagement syndrome across attention deficit/hyperactivity disorder, autism spectrum disorder, and internalizing disorders: towards a transdiagnostic construct
Source: Eur Child Adolesc Psychiatry. 2026 May 22;35(9):2925–37. doi: 10.1007/s00787-026-03058-8 (PMC13695636; doi:10.1007/s00787-026-03058-8)
Supplement: Supplementary file 1 [file 787_2026_3058_MOESM1_ESM.docx]

**Supplementary materials (“Cognitive Disengagement Syndrome across Attention Deficit/Hyperactivity Disorder, Autism Spectrum Disorder, and Internalizing Disorders: towards a transdiagnostic construct”)**

**Supplementary Tables S1 – S4 and Figures S1 – S2**

**Table S1**

*Independent t-tests for CBCL-CDS-4 by diagnosis group, sex, age range, per sample.*

| **Diagnosis group** | **Sample** | **Variable** | **Female** | | | **Male** | | | **t (gl)** | **p (2-tailed)** | **Levene’s F (p)** | **Mean diff. [95% CI]** | **Cohen’s d [95% CI]** |
| --- | --- | --- | --- | --- | --- | --- | --- | --- | --- | --- | --- | --- | --- |
|  |  |  | **N** | **M** | **(SD)** | **N** | **M** | **(SD)** |  |  |  |  |  |
|  |  |  |  |  |  |  |  |  |  |  |  |  |  |
| Internalizing disorders | No comorb. | CBCL-CDS-4 | 16 | 3.7 | (2.3) | 14 | 1.1 | (1.7) | 2.7 (28) | **.012** | 2.7 (.11) | 2.0 [0.5, 3.6] | 1.0 [0.2, 1.7] |
|  |  |  |  |  |  |  |  |  |  |  |  |  |  |
| **Diagnosis group** | **Sample** | **Variable** | **Age 6–11** | | | **Age 12–17** | | | **t (gl)** | **p (2-tailed)** | **Levene’s F (p)** | **Mean diff. [95% CI]** | **Cohen’s d [95% CI]** |
|  |  |  | **N** | **M** | **(SD)** | **N** | **M** | **(SD)** |  |  |  |  |  |
|  |  |  |  |  |  |  |  |  |  |  |  |  |  |
| ADHD | Full sample | CBCL-CDS-4 | 229 | 1.6 | (1.4) | 124 | 2.2 | (1.8) | -3.5 (206.2) | **<.001** | 13.0 (<.001) | -0.6 [-1.0, -0.3] | -0.4 [-0.6, -0.2] |
|  | No comorbidity | CBCL-CDS-4 | 82 | 1.7 | (1.4) | 46 | 2.4 | (1.8) | -2.3 (75.7) | **.027** | 4.6 (.034) | -0.7 [-1.3, -0.1] | -0.4 [-0.8, -0.1] |
| ASD | Full | CBCL-CDS-4 | 292 | 2.3 | (1.7) | 202 | 3.1 | (1.8) | -4.7 (411.2) | **<.001** | 1.2 (.281) | -0.8 [-1.1, -0.5] | -0.4 [-0.6, -0.3] |
|  | No comorbidity | CBCL-CDS-4 | 91 | 2.4 | (1.9) | 47 | 3.1 | (1.6) | -2.1 (108.5) | **.037** | 2.8 (.098) | -0.6 [-1.2, -0.0] | -0.4 [-0.7, -0.0] |
| Internalizing disorders | Full | CBCL-CDS-4 | 52 | 2.1 | (1.7) | 59 | 2.9 | (2.0) | -2.3 (109) | **.024** | 1.8 (.183) | -0.8 [-1.5, -0.1] | -0.4 [-0.8, -0.1] |
|  |  |  |  |  |  |  |  |  |  |  |  |  |  |

| **Table S2.**  *Means (M) and Standard Deviations (SD) of Study Variables by Diagnosis Group and Welch’s ANOVA Results.* | | | | | | | | | | | | | |
| --- | --- | --- | --- | --- | --- | --- | --- | --- | --- | --- | --- | --- | --- |
| **Variable** | **Full sample** | | | | | |  | **No comorbidity sample** | | | | | |
|  | **ADHD**  **(n = 353)**  **M (SD)** | **ASD**  **(n = 494)**  **M (SD)** | **Internalizing disorders**  **(n = 111)**  **M (SD)** | **Welch’s F**  **(df1, df2)** | **p** | **η²** |  | **ADHD**  **(n = 128)**  **M (SD)** | **ASD**  **(n = 138)**  **M (SD)** | **Internalizing disorders**  **(n = 30)**  **M (SD)** | **Welch’s F**  **(df1, df2)** | **p** | **η²** |
|  |  |  |  |  |  |  |  |  |  |  |  |  |  |
| CBCL-CDS-4 | 1.8 (1.6) | 2.7 (1.8) | 2.6 (1.9) | 27.4 (2, 300.6) | **<.001** | .051 |  | 1.9 (1.6) | 2.6 (1.8) | 2.8 (2.3) | 7.2 (2, 76.6) | **.001** | .046 |
| CBCL-CDS-3 | 1.5 (1.3) | 2.1 (1.5) | 2.0 (1.6) | 20.2 (2, 296.5) | **<.001** | .038 |  | 1.6 (1.3) | 2.1 (1.5) | 2.2 (1.8) | 5.2 (2, 76.9) | **.008** | .033 |
|  |  |  |  |  |  |  |  |  |  |  |  |  |  |
| CBCL item 13 – Confused or unclear thinking | 0.3 (0.5) | 0.5 (0.6) | 0.5 (0.7) | 13.1 (2, 294.5) | **<.001** | .024 |  | 0.3 (0.6) | 0.5 (0.7) | 0.5 (0.7) | 4.3 (2, 79.4) | **.017** | .027 |
| CBCL item 17 – Daydreaming or lost in thought | 0.9 (0.8) | 1.0 (0.8) | 1.0 (0.8) | 4.1 (2, 307.8) | **.017** | .009 |  | 1.0 (0.8) | 1.0 (0.8) | 0.9 (0.8) | 0.4 (2, 81.3) | .701 | .003 |
| CBCL item 80 – Stares blankly | 0.3 (0.5) | 0.6 (0.7) | 0.6 (0.7) | 22.9 (2, 295.5) | **<.001** | .040 |  | 0.3 (0.5) | 0.6 (0.7) | 0.7 (0.7) | 11.1 (2, 77.1) | **<.001** | .069 |
| CBCL item 102 – Not very active / moves slowly / low energy | 0.3 (0.6) | 0.6 (0.7) | 0.5 (0.7) | 15.3 (2, 305.8) | **<.001** | .028 |  | 0.3 (0.6) | 0.6 (0.7) | 0.6 (0.8) | 4.9 (2, 78.5) | **.010** | .032 |
|  |  |  |  |  |  |  |  |  |  |  |  |  |  |
| *Note.* Welch’s ANOVA was used due to violations of homogeneity of variances (Levene’s test p < .05 for all items except “Daydreaming or lost in thought”). Effect sizes are reported as η² (partial eta squared). | | | | | | | | | | | | | |

| **Table S3.**  *Games-Howell Post Hoc Comparisons Between Diagnosis Groups.* | | | | | | | | | | |
| --- | --- | --- | --- | --- | --- | --- | --- | --- | --- | --- |
| **Variable** | **Group Comparison** | **Full sample** | | | |  | **No comorbidity sample** | | | |
|  |  | **Mean**  **Difference**  **(I–J)** | **SE** | **95% CI**  **[LL, UL]** | **p** |  | **Mean**  **Difference**  **(I–J)** | **SE** | **95% CI**  **[LL, UL]** | **p** |
|  |  |  |  |  |  |  |  |  |  |  |
| **CBCL-CDS-4** | ASD – ADHD | 0.8 | 0.1 | 0.6 – 1.1 | **<.001** |  | 0.7 | 0.2 | 0.3 – 1.2 | **.001** |
|  | Internalizing – ADHD | 0.8 | 0.2 | 0.3 – 1.2 | **<.001** |  |  |  |  |  |
| **CBCL-CDS-3** | ASD – ADHD | 0.6 | 0.1 | 0.4 – 0.8 | **<.001** |  | 0.5 | 0.2 | 0.1 – 0.9 | **.007** |
|  | Internalizing – ADHD | 0.6 | 0.2 | 0.2 – 1.0 | **.002** |  |  |  |  |  |
| CBCL item 13 – Confused or unclear thinking | ASD – ADHD | 0.2 | 0.0 | 0.1 – 0.3 | **<.001** |  | 0.2 | 0.1 | 0.0 – 0.4 | **.025** |
|  | Internalizing – ADHD | 0.2 | 0.1 | 0.1 – 0.4 | **.005** |  |  |  |  |  |
| CBCL item 17 – Daydreaming or lost in thought | ASD – ADHD | 0.2 | 0.1 | 0.0 – 0.3 | **.012** |  |  |  |  |  |
| CBCL item 80 – Stares blankly | ASD – ADHD | 0.3 | 0.0 | 0.2 – 0.4 | **<.001** |  | 0.3 | 0.1 | 0.1 – 0.5 | **<.001** |
|  | Internalizing – ADHD | 0.3 | 0.1 | 0.1 – 0.5 | **<.001** |  | 0.5 | 0.1 | 0.1 – 0.8 | **.006** |
| CBCL item 102 – Not very active / moves slowly / low energy | ASD – ADHD | 0.3 | 0.0 | 0.1 – 0.4 | **<.001** |  | 0.2 | 0.1 | 0.0 – 0.4 | **.012** |
|  |  |  |  |  |  |  |  |  |  |  |

| **Table S4.**  *Steiger’s tests comparing overlapping correlations of CBCL-CDS items with DSM-oriented scales (N = 958).* | | | | | | |
| --- | --- | --- | --- | --- | --- | --- |
| **Item** | **Comparison** | **r**  **(first)** | **r**  **(second)** | **Δr** | **z** | **p** |
|  |  |  |  |  |  |  |
| Item 13 – Be Confused, unclear thinking | Affective vs ADHD | .30 | .15 | .15 | 3.58 | **< .001** |
|  | Affective vs ADHD_I | .30 | .21 | .09 | 2.32 | **.020** |
|  | Affective vs ADHD_H | .30 | .08 | .22 | 5.05 | **< .001** |
|  | Anxiety vs ADHD | .26 | .15 | .10 | 2.34 | **.019** |
|  | Anxiety vs ADHD_I | .26 | .21 | .05 | 1.10 | .270 |
|  | Anxiety vs ADHD_H | .26 | .08 | .17 | 3.85 | **< .001** |
|  | ASD vs ADHD | .32 | .15 | .17 | 4.30 | **< .001** |
|  | ASD vs ADHD_I | .32 | .21 | .12 | 2.91 | **.004** |
|  | ASD vs ADHD_H | .32 | .08 | .24 | 5.91 | **< .001** |
|  | ASD vs Affective | .32 | .30 | .02 | 0.68 | .494 |
|  | ASD vs Anxiety | .32 | .26 | .07 | 2.38 | **.017** |
|  |  |  |  |  |  |  |
| Item 17 – Daydreaming, lost in thought | Affective vs ADHD | .19 | .16 | .03 | 0.77 | .439 |
|  | Affective vs ADHD_I | .19 | .24 | −.05 | −1.11 | .268 |
|  | Affective vs ADHD_H | .19 | .07 | .12 | 2.71 | **.007** |
|  | Anxiety vs ADHD | .17 | .16 | .01 | 0.27 | .785 |
|  | Anxiety vs ADHD_I | .17 | .24 | −.07 | −1.49 | .136 |
|  | Anxiety vs ADHD_H | .17 | .07 | .10 | 2.17 | **.030** |
|  | ASD vs ADHD | .27 | .16 | .10 | 2.57 | **.010** |
|  | ASD vs ADHD_I | .27 | .24 | .03 | 0.64 | .524 |
|  | ASD vs ADHD_H | .27 | .07 | .19 | 4.61 | **< .001** |
|  | ASD vs Affective | .27 | .19 | .07 | 2.03 | **.042** |
|  | ASD vs Anxiety | .27 | .17 | .09 | 3.12 | **.002** |
|  |  |  |  |  |  |  |
| Item 80 – Stares blankly | Affective vs ADHD | .36 | .09 | .28 | 6.66 | **< .001** |
|  | Affective vs ADHD_I | .36 | .13 | .23 | 5.63 | **< .001** |
|  | Affective vs ADHD_H | .36 | .03 | .33 | 7.65 | **< .001** |
|  | Anxiety vs ADHD | .29 | .09 | .21 | 4.58 | **< .001** |
|  | Anxiety vs ADHD_I | .29 | .13 | .16 | 3.51 | **< .001** |
|  | Anxiety vs ADHD_H | .29 | .03 | .26 | 5.70 | **< .001** |
|  | ASD vs ADHD | .36 | .09 | .27 | 6.72 | **< .001** |
|  | ASD vs ADHD_I | .36 | .13 | .22 | 5.45 | **< .001** |
|  | ASD vs ADHD_H | .36 | .03 | .32 | 7.88 | **< .001** |
|  | ASD vs Affective | .36 | .36 | −.01 | −0.20 | .842 |
|  | ASD vs Anxiety | .36 | .29 | .07 | 2.31 | **.021** |
|  |  |  |  |  |  |  |
| Item 102 – Low energy | Affective vs ADHD | .43 | −.14 | .57 | 13.67 | **< .001** |
|  | Affective vs ADHD_I | .43 | .02 | .42 | 10.28 | **< .001** |
|  | Affective vs ADHD_H | .43 | −.22 | .65 | 15.14 | **< .001** |
|  | Anxiety vs ADHD | .25 | −.14 | .39 | 8.66 | **< .001** |
|  | Anxiety vs ADHD_I | .25 | .02 | .24 | 5.30 | **< .001** |
|  | Anxiety vs ADHD_H | .25 | −.22 | .47 | 10.38 | **< .001** |
|  | ASD vs ADHD | .25 | −.14 | .39 | 9.39 | **< .001** |
|  | ASD vs ADHD_I | .25 | .02 | .24 | 5.62 | **< .001** |
|  | ASD vs ADHD_H | .25 | −.22 | .47 | 11.18 | **< .001** |
|  | ASD vs Affective | .25 | .43 | −.18 | −5.37 | **< .001** |
|  | ASD vs Anxiety | .25 | .25 | −.00 | −0.12 | .904 |
|  |  |  |  |  |  |  |
| CDS (3 items) | Affective vs ADHD | .38 | .18 | .20 | 4.84 | **< .001** |
|  | Affective vs ADHD_I | .38 | .27 | .22 | 2.87 | **0.004** |
|  | Affective vs ADHD_H | .38 | .08 | .20 | 6.90 | **< .001** |
|  | Anxiety vs ADHD | .32 | .18 | .13 | 3.13 | **0.002** |
|  | Anxiety vs ADHD_I | .32 | .27 | .05 | 1.21 | 0.223 |
|  | Anxiety vs ADHD_H | .32 | .08 | .23 | 5.23 | **< .001** |
|  | ASD vs ADHD | .42 | .18 | .24 | 6.21 | **< .001** |
|  | ASD vs ADHD_I | .42 | .27 | .16 | 4.05 | **< .001** |
|  | ASD vs ADHD_H | .42 | .08 | .34 | 8.45 | **< .001** |
|  |  |  |  |  |  |  |

**Figure S1.**

*No comorbidity sample Welch’s ANOVA.*

Note. Boxplots of CBCL-CDS-4 scores across the three diagnostic groups (ADHD, ASD, and Internalizing disorders). Red diamonds represent group means, while thick black lines within boxes denote medians. Boxes illustrate interquartile ranges (IQR), and whiskers indicate data ranges excluding outliers. Group comparisons were conducted using Welch’s ANOVA (p < .001 for both panels), followed by Games-Howell post hoc tests.

**** p < .0001; *** p < .001; ** p < .01; * p < .05; ns = not significant.

**Figure S2.**

*No comorbidity sample Welch’s ANOVA single CBCL-CDS items.*

Note. Boxplots of CBCL-CDS items scores across the three diagnostic groups (ADHD, ASD, and Internalizing disorders). Red diamonds represent group means, while thick black lines within boxes denote medians. Boxes illustrate interquartile ranges (IQR), and whiskers indicate data ranges excluding outliers. Group comparisons were conducted using Welch’s ANOVA, followed by Games-Howell post hoc tests.

: **** p < .0001; *** p < .001; ** p < .01; * p < .05; ns = not significant.
